# Emergence of the arterial worm *Elaeophora schneideri* in moose (*Alces alces*) and tabanid fly vectors in northeastern Minnesota, USA

**DOI:** 10.1186/s13071-018-3077-0

**Published:** 2018-09-10

**Authors:** Caroline M. Grunenwald, Erika Butler, Arno Wünschmann, Anibal G. Armien, Michelle Carstensen, Erik Hildebrand, Roger D. Moon, Richard W. Gerhold

**Affiliations:** 1 Department of Microbiology, University of Tennessee, M409 Walters Life Sciences, Knoxville, TN 37996-0845 USA; 2 Nor-West Animal Clinic, 411 McIrvine Rd, Fort Frances, Ontario P9A3X7 Canada; 3 Department of Veterinary Population Medicine, Veterinary Diagnostic Laboratory, College of Veterinary Medicine, University of Minnesota, 1333 Gortner Ave, St. Paul, Minnesota 55108 USA; 4 Wildlife Health Program, Minnesota Department of Natural Resources, 5463C West Broadway, Forest Lake, Minnesota 55025 USA; 5 Department of Entomology, University of Minnesota, 219 Hodson H, 1980 Folwell Avenue, St. Paul, Minnesota 55108 USA; 6 Department of Biomedical and Diagnostic Sciences, College of Veterinary Medicine, University of Tennessee, 2407 River Drive, Room A205, Knoxville, Tennessee 37996 USA

**Keywords:** Moose (*Alces alces*), *Parelaphostrongylus tenuis*, *Elaeophora schneideri*, Parasitic infections

## Abstract

**Background:**

Moose (*Alces alces*) are a culturally and economically valued species in Minnesota. However, the moose population has experienced a sudden, marked decline in their range, including extirpation in the northwest and a 66% decline in the last decade in the northeast portions of the state. Although the exact cause of this decline is unclear, parasitic metastrongylid and filarioid nematode infections are known causes of morbidity and mortality in moose across North America.

**Methods:**

To determine if these parasitic nematodes could be contributing to the Minnesota moose population decline, we molecularly examined banked tissues obtained from moose that died of known and unknown causes for the presence of nematode DNA. Extracted brain DNA of 34 individual moose was amplified utilizing primers targeting the *18S* rRNA gene and internal transcribed spacer regions of nematodes.

**Results:**

DNA sequencing revealed that PCR products obtained from 15 (44.1%) of the moose were 99% identical to *Parelaphostrongylus tenuis*, a metastrongylid known to cause neurological disease and death. Additionally, brain tissue from 20 (58.8%) individuals yielded sequences that most closely aligned with *Elaeophora schneideri*, a parasite associated with neurological impairment but previously unreported in Minnesota. *Setaria yehi*, a common filarioid parasite of deer, was also detected in the brain tissue of 5 (14.7%) moose. Molecular screening of 618 captured tabanid flies from four trapping sites revealed *E. schneideri* was present (6%) in the Minnesota environment and transmission could occur locally. Prevalence rates among the flies ranged between 0–100% per trapping site, with *Chrysops* spp. and *Hybomitra* spp. implicated as the vectors*.*

**Conclusions:**

Ultimately, these data confirm that *P. tenuis* is widespread in the Minnesota moose population and raises the question of the significance of *E. schneideri* as a contributing factor to morbidity and mortality in moose.

**Electronic supplementary material:**

The online version of this article (10.1186/s13071-018-3077-0) contains supplementary material, which is available to authorized users.

## Background

The moose (*Alces alces*) population in Minnesota has exhibited a recent, rapid decline, raising concerns for the future of the species in the state. Between the mid 1980’s and early 2000’s, the northwestern moose population collapsed from > 4000 moose to less than 100 animals [1]. Similarly, the northeast population has decreased by approximately 66%, from 8840 animals in 2006 to 3030 in 2018 [2]. Predation, climate change, habitat alternation and disease, particularly from parasites, have all been implicated as factors contributing to the decline [3–7].

Nematode parasites, particularly lungworms (*Dictyocaulus* spp., *Protostrongylus* spp.) and filarioids (Onchocercidae spp.), are known to cause morbidity and mortality in moose and other cervids. A recent study of Minnesota moose carcasses that died from unknown causes or were euthanized due to perceived illness found that 45% of the animals had lesions consistent with nematode neural migration within central nervous system (CNS) tissues [7]. Based on histological appearance, the authors concluded the pathogenic nematode species was *Parelaphostrongylus tenuis* (Metastrongyloidea: Protostrongylidae), a common nematode parasite of white-tailed deer (*Odocoileus virginianus*) distributed throughout the eastern USA [8]. Infections by *P. tenuis* in atypical hosts, including moose, can result in severe neurological disease and mortality due to an aberrant migration of the nematode through CNS tissues [9–14].

*Parelaphostrongylus tenuis* infections are common among cervids of Minnesota [15–17]; however, with the sudden decline in moose numbers, it is possible a newly introduced pathogen is circulating in the moose population. Recent observations have raised suspicions that *Elaeophora schneideri* (Spirurida: Onchocercidae), another pathogenic nematode of moose [18–20], may be present in the Minnesota cervid population. Reports of white-tailed deer in Minnesota exhibiting facial swellings consistent with oral food impactions and cropped ears (E. Butler, personal communication) are similar to what have been previously described in *E. schneideri-*infected deer [21]. In addition, there were multiple reports of moose in the northeast region of the state with poor antler development or impaired vision due to unknown causes (E. Butler, personal communication) but consistent with clinical signs and lesions previously observed in moose infected with *E. schneideri* [18]. Historically, this parasite was thought to be limited to the western half of North America and small pockets of the southeastern USA; however, it is possible the range of *E. schneideri* has extended into Minnesota, which could explain the lesions observed in deer and moose.

The life-cycle of *E. schneideri* involves a cervid host and a hematophagous insect vector. Mule deer (*Odocoileus hemionus*) and black-tailed deer (*Odocoileus hemionus columbianus*) are definitive hosts [22–25], and transmission occurs *via* the bite of a tabanid horse fly or deer fly (Diptera: Tabanidae) [26, 27]. Tabanid flies become infected when they ingest microfilariae (L1) in a blood meal. After metamorphosis in an infected fly, third-stage larvae (L3) migrate from the fly mouthparts into the host’s circulatory system, where they eventually migrate to the carotid or leptomeningeal arteries and mature into adults (L5) [27–29]. Microfilariae progeny generally reside within the smaller capillaries of the head and neck, where they can be acquired by a feeding tabanid fly to complete the parasitic life-cycle [23].

Although infections in definitive mule deer and black-tailed deer hosts are generally subclinical, atypical hosts, including moose, white-tailed deer, elk (*Cervus canadensis*), sheep (*Ovis* spp.) and goats (*Capra hircus*), can develop elaeophorosis [25]. Elaeophorosis is characterized by obstructed blood flow, endothelial damage, thrombosis, and infarction due to the presence of nematodes in the carotid and cephalic arterial system [18, 30, 31]. This disruption in arterial circulation can lead to blindness; ischemic necrosis of the brain, ears, muzzle and other cephalic tissues; poor antler development; oral food impactions; and death [18–21, 30, 32].

We investigated the potential for non-endemic parasitic nematodes, particularly *E. schneideri*, to be associated with CNS disease in Minnesota moose. Our objective was to determine if filarioid parasites were detectable in CNS tissue samples of moose from Wünschmann et al. [7] and tabanid flies in Minnesota. Our data reveal that *E. schneideri* is indeed present in the Minnesota moose herd and tabanid horsefly vectors, suggesting the parasite could contribute to morbidity and mortality in moose and transmission can occur locally. This study serves as the first documentation of *E. schneideri* in Minnesota and the Midwest, as well as provides new insight into the potential causes of morbidity and mortality in the Minnesota moose population with implications for future population management.

## Methods

### Moose sample collection and tissue histology

Carcasses from 62 Minnesota moose were necropsied and CNS tissues examined histologically for any pathological changes [7]. Briefly, tissues were collected from carcasses of moose that died of unknown causes, vehicular collisions, or were euthanized by tribal or Department of Natural Resources personnel due to various clinical signs. Sections of central nervous system (CNS) tissues were preserved in 10% neutral buffered formalin, embedded in paraffin, sectioned, and stained with H&E for microscopic examination. Specimens were categorized as histologically positive (HP) if migration tracts, larvae, morulae, or cross-sections of adult nematodes were visible, whereas specimens with no pathological changes evident in the CNS were categorized as histologically negative (HN). Based on these results, 34 animals (22 HP; 12 HN) were selected for further molecular analysis.

### Molecular testing of moose tissues

To screen the preserved moose CNS tissues for the presence of pathogenic nematodes, two separate 10 μm shavings were obtained from the formalin-fixed, paraffin-embedded tissue blocks for molecular analysis. Duplicate shavings were examined to increase sensitivity. Each shaving was subjected to DNA extraction according to manufacturer’s instructions (DNEasy Blood & Tissue Kit, Qiagen, Valencia, CA, USA). DNA extraction control was included to detect contamination during the DNA extraction process. Purified DNA was first screened for the presence of *P. tenuis* using a nested polymerase chain reaction (PCR) with the primary primer pair PTP1 (5'-CCG TCG AAT ACA TGT CAT CC-3') and PTP2 (5'-TCG TCA AGA CGA TGA TTC CC-3'); and the secondary primer pair PtIntITSF (5'-AGA ATT ACG ACA ATG GCA AC-3') and PtIntITSR (5'-ATG ATA CCC ATT GAT AAT C-3'), as previously described [13, 14, 33]. This assay is designed to selectively amplify a 110 bp portion of the second internal transcribed spacer region (ITS2) of *Parelaphostrongylus* spp. [33]. To screen for the presence of other nematodes in the moose CNS tissues, the Nematoda-wide primers Nem18SF (5'-CGC GAA TRG CTC ATT ACA ACA GC-3') and Nem18SR (5'-GGG CGG TAT CTG ATC GCC-3') that targets a 508 bp segment of the *18S* rRNA gene were utilized as previously described [34]. All positive PCR products were purified using the Qiagen PCR Purification Kit (Qiagen, Valencia, CA, USA) and sequenced at the University of Tennessee Genomics Core (Knoxville, TN, USA).

### Tabanid fly collection and molecular screening

**Fig. 1:**
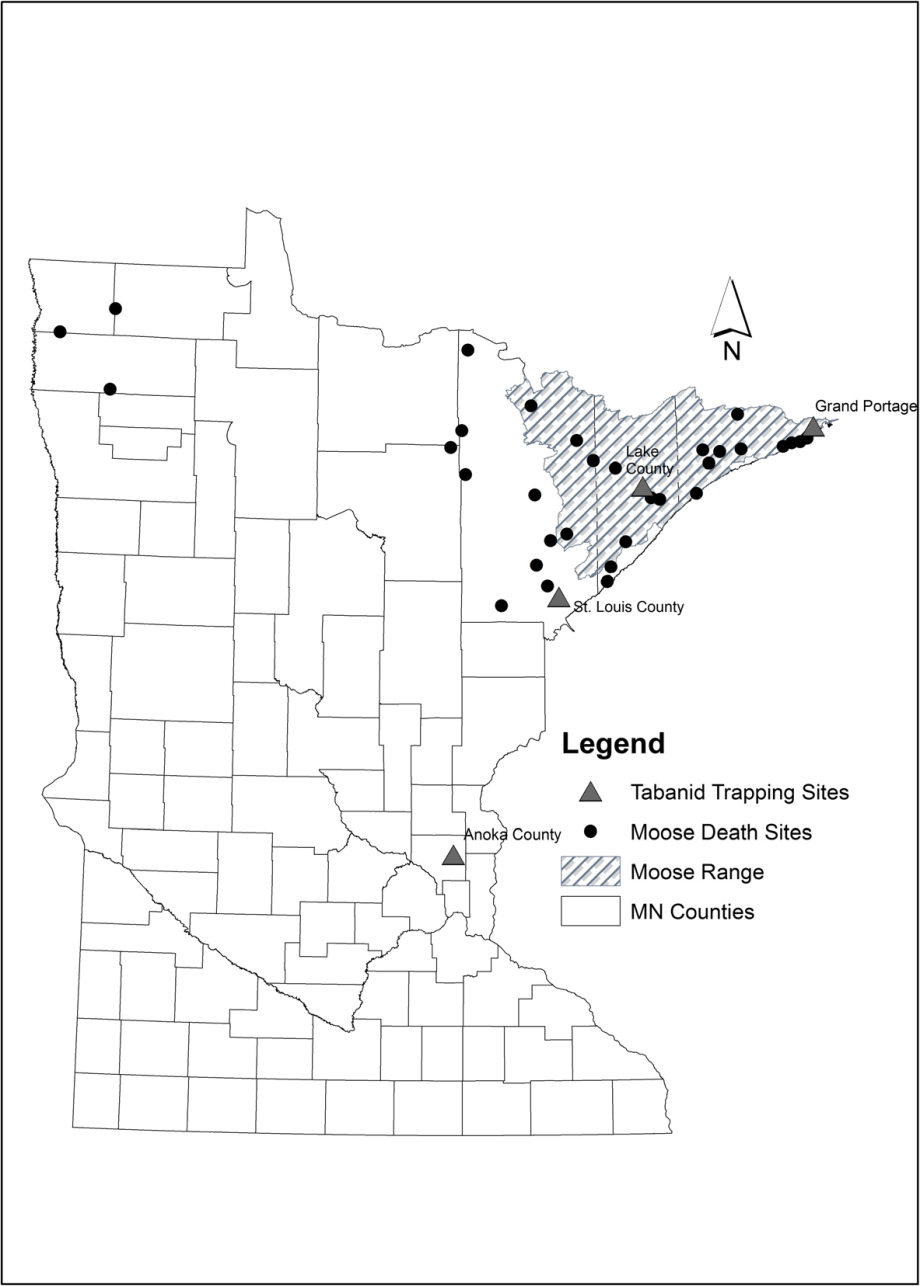
Geographical location of tabanid fly trapping sites and MN moose mortality sites. Four individual trapping sites were chosen for fly collection during June-August of 2013. Sites of moose mortalities are marked with circles. The shaded region represents the current MN moose range

To survey for the presence of cervid nematode parasites in tabanid flies, 618 flies were collected from four locations in Minnesota (Fig. 1) using a canopy trap [35]. Sites were chosen based on their proximity to known moose habitat, remoteness and ease of access. Three of the four sites fall within the current Minnesota moose range and the fourth site, located in the Carlos Avery Wildlife Management Area in Anoka County, serves as an outside representative. Traps were operated intermittently in June-September of 2013. Captured specimens were stored at ~20 °C until they could be sorted, identified to genus, and then preserved in 70% ethanol for later DNA extraction. DNA was extracted using the Qiagen DNeasy Blood and Tissue Kit (Qiagen, Valencia, CA, USA) following manufacturer's instructions. Flies were divided into groups of 10, and 5 μl of DNA from each fly was pooled into the corresponding group’s microcentrifuge tube. To screen for nematode DNA within pooled fly DNA, a PCR reaction with the Nem18S primers described above were utilized. Individual flies in PCR-positive DNA pools were subjected to an additional PCR reaction using the same Nem18S primers. All positive reactions were purified using the Qiagen PCR Purification Kit (Qiagen, Valencia, CA, USA) and sequenced at the University of Tennessee’s Genomics Core (Knoxville, TN, USA).

### Phylogenetic analysis of parasite sequences

All *18S* and ITS2 consensus sequence chromatograms were trimmed and edited by hand using Sequencher 5.3 (Gene Codes Corporation, Ann Arbor, MI, USA). Edited sequences were compared against the NCBI GenBank database. Due to the scarcity of published genetic data for parasitic nematodes known to infect cervids, we also compared our genetic data with sequences obtained from adult reference nematodes (Additional file 1: Table S1) that we identified morphologically and subjected to DNA extraction and PCR amplification as described above. Alignment and construction of neighbor-joining trees of *18S* nematode sequences were done using MEGA 6.0 [36]. All consensus sequences were deposited in the GenBank database under the accession numbers KT020850, KT031393, KT878970, KT878971, KT878974, KT878980-KT878988, KT885226, KT885227, KT907501-KT907509 and KT93494.

### Parasite prevalence estimates and statistical analysis

To estimate the prevalence of infected fly vectors, we compared the percentage of infected flies with each nematode species that was identified through DNA sequencing and phylogenetics. Our analysis also included a comparison of parasite prevalence among trapping sites, as well as among different fly genera using Dunn’s multiple comparisons test (*P* ≤ 0.05). Statistical analyses were performed with GraphPad Prism v.6 (GraphPad Software, La Jolla, CA, USA).

## Results

### Multiple nematode species detected in CNS tissues of free-ranging Minnesota moose

Using Nematoda-wide *18S* primers, nematode DNA was successfully amplified and sequenced from 24 (68.6%) individual moose. When compared against GenBank, sequences most closely aligned with *18S* sequences from nematode species of the family Onchocercidae, including *Setaria digitata* (GenBank: DQ094175.1), with a 98–99% maximum identity. Despite the high percent identity, *S. digitata* is not commonly associated with cervids and is not endemic to North America [25, 37–39]. Due to the highly conserved nature of the *18S* rRNA target, we concluded the sequences from the Minnesota moose tissues indeed belonged to filarioid nematodes; however, they were most likely not *S. digitata*.

**Fig. 2:**
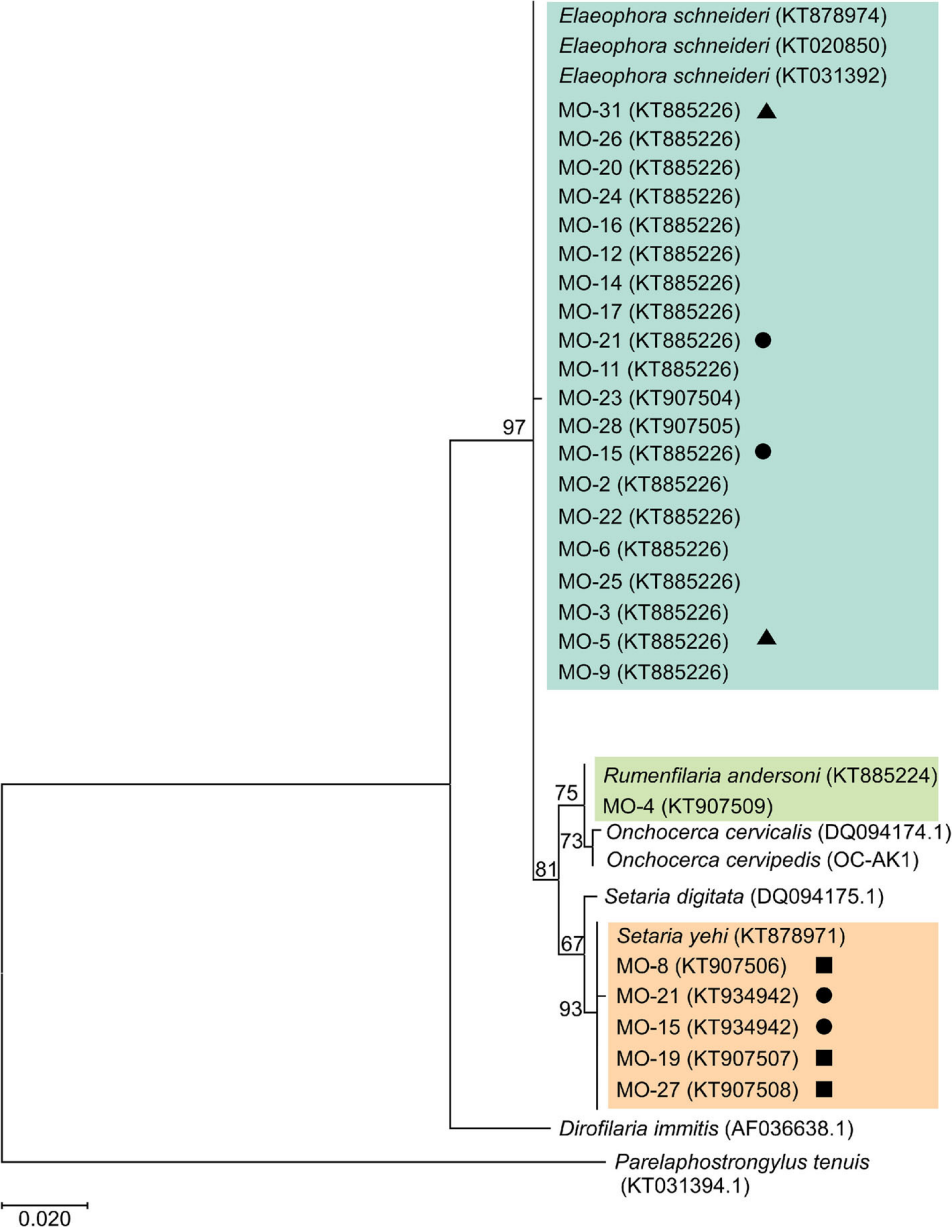
Phylogenetic analysis of partial nematode*18S* sequences (508 bp) obtained from formalin-fixed paraffin-embedded CNS tissues of various ruminants. Tree was constructed using the maximum likelihood method and the evolutionary distances computed using the Kimura 2-parameter method. Bootstrap values ≥ 50% are shown above the branches. The tree is drawn to scale. Reference nematodes are labeled with their respective NCBI GenBank accession number. Markers indicate the detection of double infections (triangles, *P. tenuis* + *E. schneideri*; circles, *S. yehi* + *E. schneideri*; squares, *P. tenuis* + *S. yehi*)

A phylogenetic comparison of *18S* sequences from the Minnesota CNS moose tissues and reference specimens revealed the presence of 3 distinct species of filarioids in the moose CNS tissue samples (Fig. 2): *Setaria yehi*, *E. schneideri* and *Rumenfilaria andersoni*. The arterial worm, *E. schneideri*, was detected in 20 individual moose, 10 (50%) of which had no histological evidence of CNS nematode infections, and 10 moose had either migration tracts or nematodes visible in the CNS sections (Table 1). A second nematode species, *S. yehi*, was detected in one histologically-negative moose and four moose with migration tracts in the CNS. Two animals were positive for *E. schneideri* and *S. yehi*. *Rumenfilaria andersoni* was detected in a single moose. All nematode sequences obtained were deposited into GenBank (Additional file 2: Table S2).

**Table 1:** Summary of demographics, histology, and sequence results for Minnesota moose CNS tissues (*n* = 34; 2004–2013). Negative histology results refer to animals with no pathological changes in the CNS tissues consistent with a nematode infection

Moose ID	Sex (M/F)	Age^a^	Histology results	*P. tenuis* (+/-)	*E. schneideri* (+/-)	*S. yehi* (+/-)	*R. andersoni* (+/-)
MO-32	F	Adult	Migration tracts present	+	-	-	-
MO-19	F	Calf	Migration tracts present	+	-	+	-
MO-27	F	Adult	Migration tracts present	+	-	+	-
MO-6	F	Adult	Migration tracts present	-	+	-	-
MO-2	M	Adult	Migration tracts present	-	+	-	-
MO-17	F	Yearling	Migration tracts present	-	+	-	-
MO-3	F	Adult	Migration tracts present	-	+	-	-
MO-25	M	Calf	Migration tracts present	-	+	-	-
MO-29	F	Adult	Migration tracts present	-	+	-	-
MO-15	F	Yearling	Migration tracts present	-	+	+	-
MO-21	M	Yearling	Migration tracts present	-	+	+	-
MO-10	F	Yearling	Migration tracts present	-	-	-	-
MO-18	F	Yearling	Morulae present	+	-	-	-
MO-7	M	Yearling	Morulae present	+	-	-	-
MO-1	F	Calf	Larvae present	+	-	-	-
MO-13	F	Adult	Larvae present	+	-	-	-
MO-4	M	Yearling	Adult, morulae present	+	-	-	+
MO-5	F	Yearling	Adult, morulae present	+	+	-	-
MO-35	F	Calf	Adult present	+	-	-	-
MO-26	M	Calf	Adult present	+	-	-	-
MO-33	F	Yearling	Adult present	+	-	-	-
MO-30	M	Adult	Adult present	+	-	-	-
MO-31	M	Adult	Adult present	+	+	-	-
MO-8	M	Yearling	Negative	+	-	+	-
MO-9	M	Yearling	Negative	-	+	-	-
MO-11	F	Adult	Negative	-	+	-	-
MO-12	F	Yearling	Negative	-	+	-	-
MO-14	F	Adult	Negative	-	+	-	-
MO-16	F	Calf	Negative	-	+	-	-
MO-20	M	Adult	Negative	-	+	-	-
MO-22	M	Adult	Negative	-	+	-	-
MO-23	F	Adult	Negative	-	+	-	-
MO-24	M	Calf	Negative	-	+	-	-
MO-28	M	Adult	Negative	-	+	-	-
Total number of moose^b^				15	20	5	1

^a^Moose were categorized as adults (> 2 years-old), yearlings (12–23 months-old), or calves (< 1 year-old)

^b^Total number of moose refers to the total number of animals that were sequence-positive for each parasite species

### *Elaeophora schneideri* present in Minnesota tabanid flies

A total of 618 tabanid flies in three genera were trapped from four locations (Table 2) and molecularly screened for the presence of filarioid nematodes. In order of descending abundance, the flies identified were *Chrysops* spp., *Hybomitra* spp. and *Tabanus* spp. Representatives from all three genera were collected at each of the trapping sites except for the St. Louis County location, where only *Chrysops* spp. were obtained.

**Table 2:** Fly counts per genus and trapping location used in the molecular survey for filarioid nematodes. Trapping locations correspond to the locations detailed in Fig. 1. Number of flies that were PCR-positive for *E. schneideri* are listed first, followed by the total fly count. Prevalence values (% PCR-positive for *E. schneideri*) are listed in parentheses

Fly genus	Anoka County	Grand Portage	Lake County	St. Louis County	Total no. of flies per genus (% prevalence)
*Chrysops* spp.	5/364 (1.4)	0/103 (0)	5/18 (27.8)	21/21 (100)	31/506 (6.1)
*Hybomitra* spp.	5/62 (8.1)	0/6 (0)	0/34 (0)	0/0 (0)	5/102 (4.9)
*Tabanus* spp.	0/7 (0)	0/1 (0)	0/2 (0)	0/0 (0)	0/10 (0)
Total no. of flies per location (% prevalence)	10/433 (2.3)	0/110 (0)	5/54 (9.3)	21/21 (100)	36/618 (5.8)

**Fig. 3:**
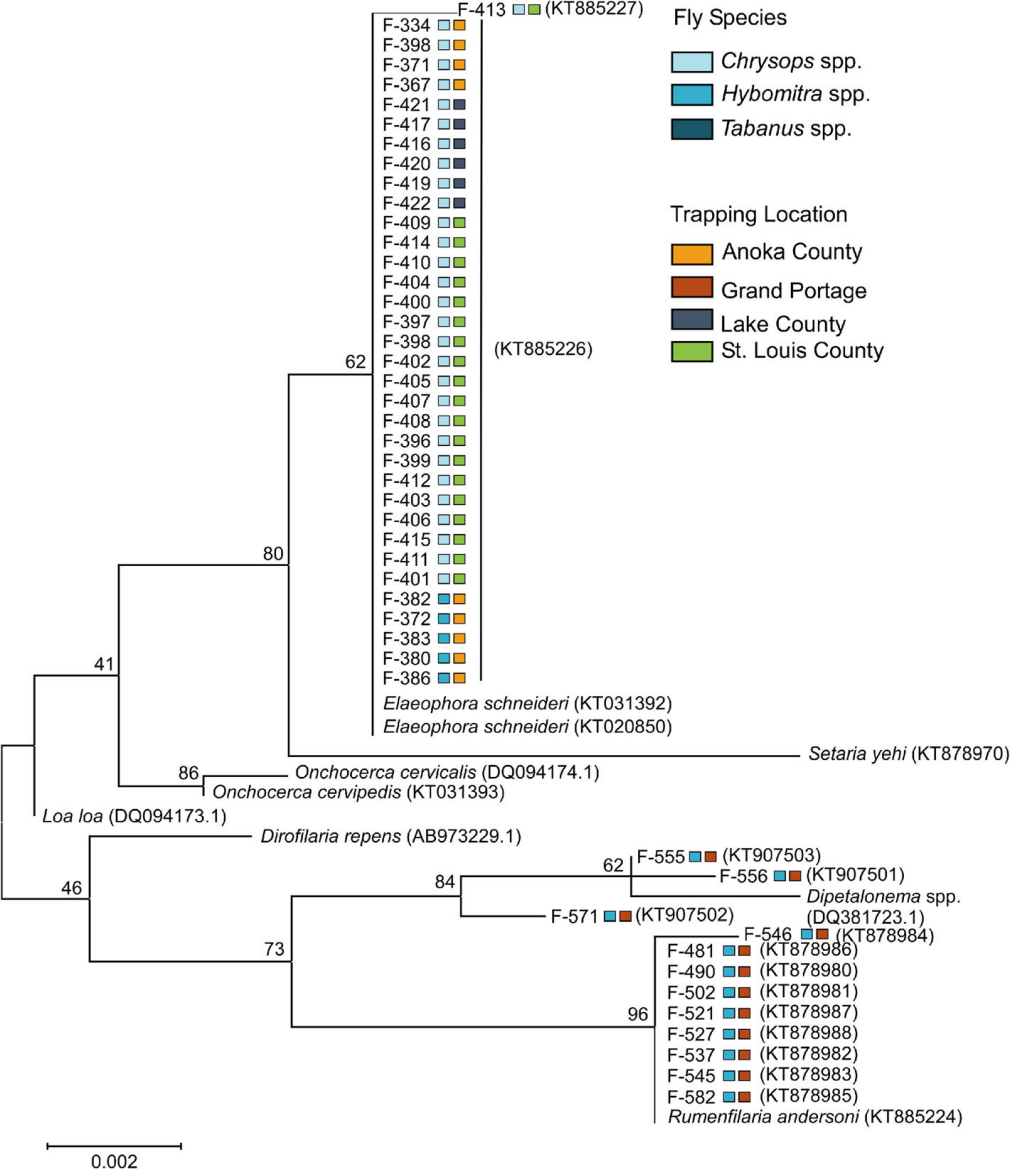
*18S* rRNA gene sequencing and phylogenetic analysis reveals the presence of multiple filarioid nematodes in Minnesota tabanid horseflies. Fifty-four nematode *18S* rRNA gene sequences (796 bp) were used in the analysis. Fly isolate F-369 was not included in the analysis due to poor quality sequence data. The evolutionary history was inferred using the maximum likelihood method and evolutionary distances computed using the Kimura 2-parameter method. The tree is drawn to scale. Bootstrap values are shown above the branches. Colored markers correspond to fly species and trapping location. Reference nematodes are labeled with their respective NCBI GenBank accession number

**Fig. 4:**
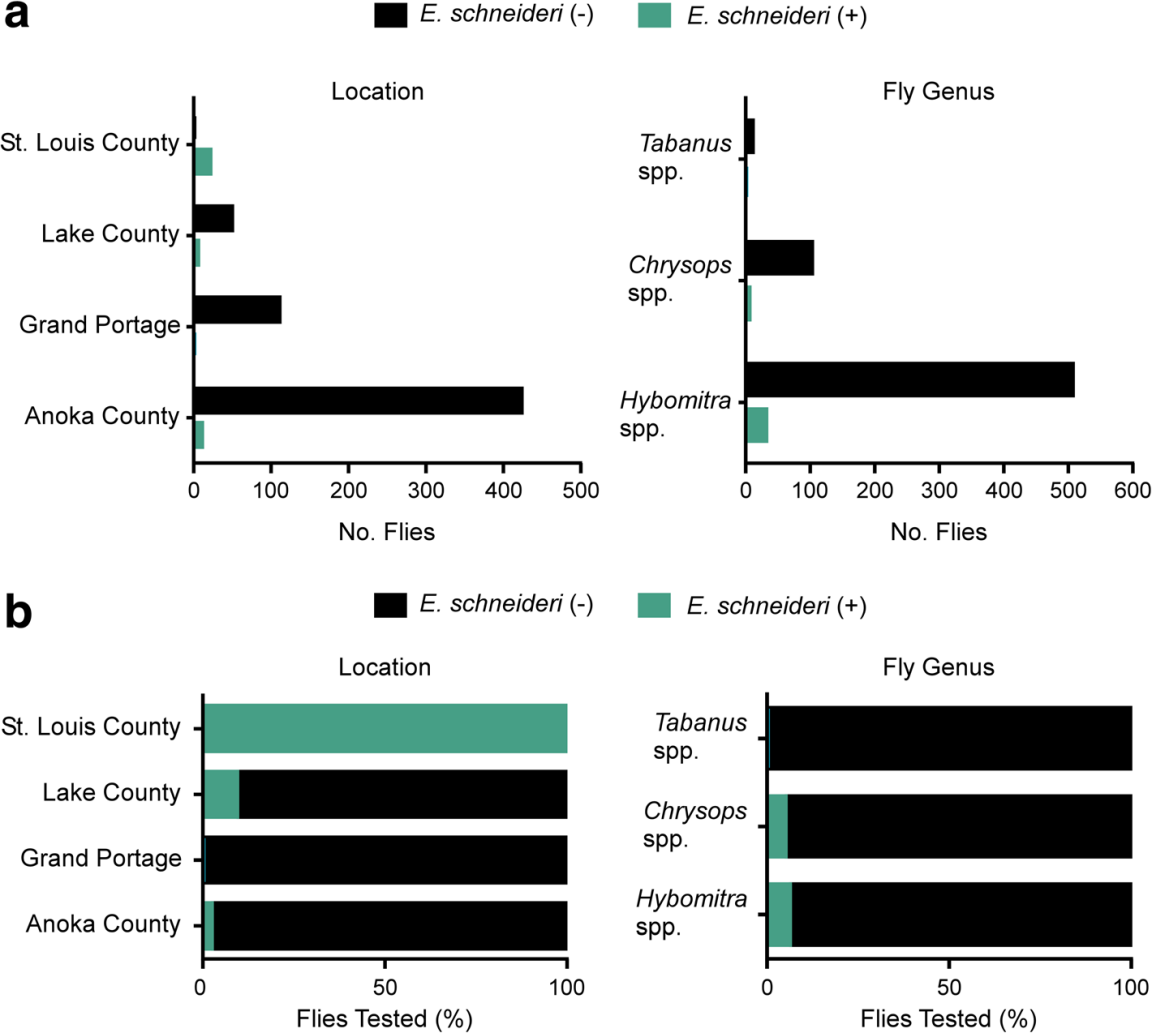
Detectable *E. schneideri* in Minnesota tabanid horseflies varies among fly species and trapping sites. Number of flies tested (**a**) and prevalence (number of positive flies / total number of flies) (**b**) that were PCR-positive (blue) or negative (black) for *E. schneideri* was determined based on *18S* sequencing results for each trapping location and each of the fly genera tested

PCR screening and sequencing with Nematoda-specific *18S* rRNA primers revealed 5.8% (95% CI: 4.0–7.6%) of the flies tested were positive for *E. schneideri* (Fig. 3)*.* The majority of *E. schneideri-*sequence-positive flies were *Chrysops* spp. (86.2%; 95% CI: 74.9–97.5%), with *Hybomitra* spp. representing only 13.9% (95% CI: 2.6–25.2%) (Fig. 4). *Elaeophora schneideri* was not detected in the *Tabanus* spp. tested. Although prevalence of *E. schneideri* varied slightly across fly genera, these differences were not statistically significant (Friedman test with Dunn’s correction for multiple comparisons, adjusted *P*-value < 0.05 used to indicate a significant difference). Prevalence of *E. schneideri* across trapping sites varied greatly, ranging from 0% (0 of 110) at Grand Portage to 2.3% (95% CI: 0.9–3.7%) at Anoka County, to 9.3% (95% CI: 1.5–17.1%) at Lake County, and 100% (21 of 21) at the St. Louis County location (Fig. 4). However, these differences were not statistically significant (Friedman test with Dunn’s correction for multiple comparisons, adjusted *P*-value > 0.05).

### Other filarioid species detected in Minnesota tabanid flies

In addition to *E. schneideri*, two other species of filarioid worms were detected in the Minnesota horse flies (Fig. 3). *Rumenfilaria andersoni* was detected in 9 *Chrysops* spp. flies from the Grand Portage site. Furthermore, additional unique filarioid sequences were detected in three *Chrysops* spp. flies. When compared against GenBank, the unknown filarioid sequences most strongly aligned with *18S* sequences from *Dipetalonema* spp. (GenBank: DQ531723.1) and *Loa loa* (GenBank: DQ094173.1) with 99% identities. Based on the sequence data, we were unable to definitively identify the genus or species of these unknown nematode(s).

### Multiple *E. schneideri 18S* haplotypes identified in hosts and vectors of Minnesota

A comparison of all *E. schneideri 18S* sequences amplified from tabanid and moose hosts revealed the presence of four distinct *18S* haplotypes (Additional file 2: Table S2, Additional file 3: Table S3). Two *18S* haplotypes were observed among the tabanid *E. schneideri* sequences, with 94% (34 of 36) representing a single haplotype we denoted as “ES-1”; the other haplotype had a single representative and was labeled as “ES-2.” The sequence quality was poor for one isolate, F-369, and was therefore not included in this analysis. Analysis of the *18S* rRNA gene sequences from the moose CNS tissues revealed 90 ± 13.2% (95% CI: 76.8–100%) of the sequences were identical to the ES-1 haplotype found in the flies. Two additional unique haplotypes, each with a single representative (“ES-3,” “ES-4”; Additional file 2: Table S2) were also observed.

## Discussion

To our knowledge, this study is the first to report the presence of the arterial worm *E. schneideri* in the Midwest of the USA, specifically Minnesota, indicating *E. schneideri* could be an emerging pathogen in moose. Historically, *E. schneideri* was thought to be primarily found in the western half of North America, presumably in conjunction with the geographical range of the well-adapted mule deer and black-tailed deer definitive hosts. In these hosts, prevalence of *E. schneideri* is high, reaching levels of 78–100% [26, 32]. However, the parasite has also been reported in white-tailed deer in Florida, Georgia, South Carolina and Texas with a much lower prevalence of 2–10% [24, 40]. It is speculated that the emergence of *E. schneideri* in the southeastern USA is a consequence of the translocation of infected deer from endemic areas in the West [25, 31, 40]. At this time, it is uncertain if the emergence of *E. schneideri* in Minnesota is due to an importation event(s) or a natural expansion of the parasite’s geographical range. Alternatively, *E. schneideri* could be endemic in Minnesota moose but remained undetected due to low prevalence. Future comparative genetics studies of various geographical isolates may provide insight into the origin of Minnesota *E. schneideri*.

Our surveillance of tabanid flies for the presence of filarioid nematodes further confirms the presence of *E. schneideri* in Minnesota and suggests the nematode is being transmitted and maintained in the environment. An overall prevalence of 5.8% in the Minnesota flies was relatively high compared to the 0.3% reported in South Carolina [41] and 0.8% in Montana [42]; however, prevalence rates as high as 20% have been reported in New Mexico [26, 43]. Interestingly, we were able to sequence *E. schneideri* from *Chrysops* spp. and *Hybomitra* spp. flies (Fig. 2). Previous surveys implicated *Hybomitra* spp. as an intermediate host for *E. schneideri* [42–44], but this is the first time the parasite has been detected in *Chrysops* spp. flies. Although we failed to detect *E. schneideri* in *Tabanus* spp. flies, we recognize our sample size was low, and thus, it remains undetermined if *Tabanus* spp. contribute to the eco-epidemiology of *E. schneideri* in the Minnesota system.

The presence of *E. schneideri* could have significant health implications for Minnesota moose. Reported clinical signs of elaeophorosis in moose include sloughed ear tips [19], blindness [18, 20], neurological impairment and death [45]. Typically, this is due to the nematodes restricting blood flow in the carotids and other cephalic arteries, leading to the development of ischemic lesions [18, 20, 30]. Interestingly, neither ischemic cerebrocortical necrosis nor the presence of intra-arterial nematodes in the brain, including meninges, were observed in any of the cases. Instead, animals with abnormal histological findings exhibited lesions consistent with the metastrongylid parasite, *P. tenuis.* All moose with observed nematodes were sequence-positive for *P. tenuis*, which is expected since this organism is a common CNS pathogen in Minnesota moose [7]. However, *E. schneideri* was detected in moose with both normal histology and lesions consistent with nematode migration tracts. Aberrant migration has not been reported with *E. schneideri*, thus the observed migration tracks are most likely caused by coinfection with *P. tenuis*. Although these samples were PCR-negative for *P. tenuis*, amplification of DNA from FFPE samples lacking visible nematodes can have limited success [46]. We suspect the *E. schneideri* detected in the Minnesota moose CNS tissues originated in the arteries, most likely of the leptomeninges, of the cranium, and may have been dislodged during necropsy. Under these conditions, the parasites may not have caused overt gross or histological lesions. This suggests infection with *E. schneideri* may not necessarily result in clinical CNS disease.

Previous studies surveying hunter-killed moose for *E. schneideri* implied elaeophorosis is relatively mild in these animals, with many exhibiting subclinical infections [45, 47]; however, both surveys took place in endemic regions (Colorado and Wyoming) and only examined seemingly healthy individuals. We would predict co-infections with *E. schneideri* and *P. tenuis* or other parasite species would negatively impact overall moose health. We speculate that these infections could lead to a compromised immune state, allowing greater susceptibility to other pathogens, predation, or result in decreased reproductive rates. In Minnesota, where the moose population is already impacted negatively by parasites, including *P. tenuis*, liver flukes (*Fascioloides magna*) and winter ticks (*Dermacentor albipictus*) [7], the emergence of *E. schneideri* could further repress an already struggling population.

In addition to *E. schneideri*, we also detected the presence of other filarioid species in the moose CNS tissues and tabanid flies. Sequences from unidentified species of filarioid nematodes were amplified from three *Chrysops* spp. flies from the Grand Portage trapping site (Fig. 2). We were unable to determine the identity or the significance, if any, of these findings. Future studies examining the flies for infectious third stage larvae may allow these species to be differentiated based on morphological characters. Screening of the CNS tissue samples revealed *S. yehi* and *R. andersoni* were present in a select number of Minnesota moose (Table 1). *Setaria yehi* is a common parasite of white-tailed deer and found in the peritoneal cavity. No associations with disease in moose have been reported, although other *Setaria* species, namely *Setaria digitata* and *Setaria cervi*, have documented neurotropism in cattle and deer, respectively [25, 48]. *Rumenfilaria andersoni*, another filarioid nematode, infects moose, caribou (*Rangifer tarandus*) and white-tailed deer [49–52]. Adult *R. andersoni* reside within the lymphatic vessels of the rumen and microfilariae can be observed in the general circulatory system [49, 50]. It is unknown if infections with *R. andersoni* can lead to clinical or subclinical disease; only macroscopic inflammatory changes within the ruminal vessels of infected reindeer have been described [51]. Microfilariae identified as *S. yehi* and *R. andersoni* have been observed in blood samples from live-captured moose of Minnesota [52], so detection of these microfilariae in these samples is not surprising.

## Conclusions

To our knowledge, this study is the first to document the presence of the pathogenic nematode *E. schneideri* in moose and tabanid fly populations of Minnesota, USA, indicating the occurrence of local transmission and expanding the current known distribution of *E. schneideri.* A better understanding of the distribution of *E. schneideri* is essential to help prevent the spread of this parasite to other non-endemic locations through human-mediated translocation of infected cervids, as well as its potentially negative economic impact on domestic farmers (loss of livestock, cost of treatment, etc.) or state and local governments (loss of hunting and ecotourism revenues). Furthermore, we were able to enhance our understanding of *E. schneideri* eco-epidemiology by implicating another genus of tabanid flies as a newly discovered vector of *E. schneideri*. These data will help set the foundation for future research investigating *E. schneideri*, particularly with regards to elaeophorosis and the potential impact it may have on moose and other cervid populations.

## Additional files

Additional file 1: **Table S1.** Reference nematodes utilized in *18S* molecular analysis. Adult nematodes were identified based on morphological characters. Geographical origin and host species refer to the place and host from which the adult nematode was isolated. (DOCX 12 kb)

Additional file 2: **Table S2.** Fiarioid *18S* rRNA sequences obtained from Minnesota moose CNS tissues. *Elaeophora schneideri* Isolates with identical *18S* sequences are assigned the same haplotype number. A representative sequence for each haplotype was deposited in the GenBank database. (DOCX 12 kb)

Additional file 3: **Table S3.** Fiarioid *18S* rRNA sequences obtained from Minnesota tabanid horseflies (*n* = 36; 2013). Isolates with identical *18S* sequences are assigned the same haplotype number. *Elaeophora schneideri* sequences with ambiguous characters are labeled with ND and were not included in the haplotype analysis. (DOCX 14 kb)

## Abbreviations

CNS: Central nervous system
HN: Histologically negative
HP: Histologically positive
ITS2: Second internal transcribed spacer region
ND: Not done
