# Supplementary material for: Emergence of the arterial worm Elaeophora schneideri in moose (Alces alces) and tabanid fly vectors in northeastern Minnesota, USA
Source: Parasit Vectors. 2018 Sep 10;11:507. doi: 10.1186/s13071-018-3077-0 (PMC6131914; doi:10.1186/s13071-018-3077-0)
Supplement: Supplementary file 1 — Table S1. Reference nematodes utilized in 18S molecular analysis. Adult nematodes were identified based on morphological characters. Geographical origin and host species refer to the place and host from which the adult nematode was isolated. (DOCX 12 kb) [file 13071_2018_3077_MOESM1_ESM.docx]

| **Isolate** | **Species** | **Geographic Origin** | **Host Species** | **GenBank Accession No.** |
| --- | --- | --- | --- | --- |
| RA-F1 | *Rumenfilaria andersoni* | Finland | *Rangifer tardanus* | KT885224 |
| ES-WY11 | *Elaeophora schneideri* | Wyoming, USA | *Alces alces* | KT031392 |
| ES-GA1 | *Elaeophora schneideri* | Georgia, USA | *Odocoileus virginianus* | KT878974 |
| ES-CA1 | *Elaeophora schneideri* | California, USA | *Rusa unicolor* | KT020850 |
| OC-AK1 | *Onchocerca cervipidis* | Alaska, USA | *Alces alces* | KT031393 |
| SY-AK1 | *Setaria yehi* | Alaska, USA | *Alces alces* | KT878970 |
| SY-GA1 | *Setaria yehi* | Georgia, USA | *Odocoileus virginianus* | KT878971 |
