# Supplementary material for: Emergence of the arterial worm Elaeophora schneideri in moose (Alces alces) and tabanid fly vectors in northeastern Minnesota, USA
Source: Parasit Vectors. 2018 Sep 10;11:507. doi: 10.1186/s13071-018-3077-0 (PMC6131914; doi:10.1186/s13071-018-3077-0)
Supplement: Supplementary file 2 — Table S2. Fiarioid 18S rRNA sequences obtained from Minnesota moose CNS tissues. Elaeophora schneideri Isolates with identical 18S sequences are assigned the same haplotype number. A representative sequence for each haplotype was deposited in the GenBank database. (DOCX 12 kb) [file 13071_2018_3077_MOESM2_ESM.docx]

| **Moose ID** | **Nematode Species** | ***E. schneideri* Haplotype** | **GenBank Accession No.** |
| --- | --- | --- | --- |
| MO-2 | *Elaeophora schneideri* | ES-1 | KT885226 |
| MO-3 | *Elaeophora schneideri* | ES-1 | KT885226 |
| MO-5 | *Elaeophora schneideri* | ES-1 | KT885226 |
| MO-6 | *Elaeophora schneideri* | ES-1 | KT885226 |
| MO-9 | *Elaeophora schneideri* | ES-1 | KT885226 |
| MO-11 | *Elaeophora schneideri* | ES-1 | KT885226 |
| MO-12 | *Elaeophora schneideri* | ES-1 | KT885226 |
| MO-14 | *Elaeophora schneideri* | ES-1 | KT885226 |
| MO-16 | *Elaeophora schneideri* | ES-1 | KT885226 |
| MO-17 | *Elaeophora schneideri* | ES-1 | KT885226 |
| MO-20 | *Elaeophora schneideri* | ES-1 | KT885226 |
| MO-22 | *Elaeophora schneideri* | ES-1 | KT885226 |
| MO-23 | *Elaeophora schneideri* | **ES-3** | KT907504 |
| MO-24 | *Elaeophora schneideri* | ES-1 | KT885226 |
| MO-25 | *Elaeophora schneideri* | ES-1 | KT885226 |
| MO-28 | *Elaeophora schneideri* | **ES-4** | KT907505 |
| MO-29 | *Elaeophora schneideri* | ES-1 | KT885226 |
| MO-31 | *Elaeophora schneideri* | ES-1 | KT885226 |
| MO-15 | *Elaeophora schneideri; Setaria yehi* | ES-1 | KT885226; KT934942 |
| MO-21 | *Elaeophora schneideri; Setaria yehi* | ES-1 | KT885226; KT934942 |
| MO-8 | *Setaria yehi* | NA | KT907506 |
| MO-19 | *Setaria yehi* | NA | KT907507 |
| MO-27 | *Setaria yehi* | NA | KT907508 |
| MO-4 | *Rumenfilaria andersoni* | NA | KT907509 |
