# Supplementary material for: Emergence of the arterial worm Elaeophora schneideri in moose (Alces alces) and tabanid fly vectors in northeastern Minnesota, USA
Source: Parasit Vectors. 2018 Sep 10;11:507. doi: 10.1186/s13071-018-3077-0 (PMC6131914; doi:10.1186/s13071-018-3077-0)
Supplement: Supplementary file 3 — Table S3. Fiarioid 18S rRNA sequences obtained from Minnesota tabanid horseflies (n = 36; 2013). Isolates with identical 18S sequences are assigned the same haplotype number. Elaeophora schneideri sequences with ambiguous characters are labeled with ND and were not included in the haplotype analysis. (DOCX 14 kb) [file 13071_2018_3077_MOESM3_ESM.docx]

| **Fly ID** | **Trapping Location** | **Fly Genus** | **Filarid Species** | ***E. schneideri* Haplotype** | **GenBank Accession No.** |
| --- | --- | --- | --- | --- | --- |
| F-396 | St. Louis County | *Chrysops* | *Elaeophora schneideri* | ES-1 | KT885226 |
| F-397 | St. Louis County | *Chrysops* | *Elaeophora schneideri* | ES-1 | KT885226 |
| F-398 | St. Louis County | *Chrysops* | *Elaeophora schneideri* | ES-1 | KT885226 |
| F-399 | St. Louis County | *Chrysops* | *Elaeophora schneideri* | ES-1 | KT885226 |
| F-400 | St. Louis County | *Chrysops* | *Elaeophora schneideri* | ES-1 | KT885226 |
| F-401 | St. Louis County | *Chrysops* | *Elaeophora schneideri* | ES-1 | KT885226 |
| F-402 | St. Louis County | *Chrysops* | *Elaeophora schneideri* | ES-1 | KT885226 |
| F-403 | St. Louis County | *Chrysops* | *Elaeophora schneideri* | ES-1 | KT885226 |
| F-404 | St. Louis County | *Chrysops* | *Elaeophora schneideri* | ES-1 | KT885226 |
| F-405 | St. Louis County | *Chrysops* | *Elaeophora schneideri* | ES-1 | KT885226 |
| F-406 | St. Louis County | *Chrysops* | *Elaeophora schneideri* | ES-1 | KT885226 |
| F-410 | St. Louis County | *Chrysops* | *Elaeophora schneideri* | ES-1 | KT885226 |
| F-408 | St. Louis County | *Chrysops* | *Elaeophora schneideri* | ES-1 | KT885226 |
| F-409 | St. Louis County | *Chrysops* | *Elaeophora schneideri* | ES-1 | KT885226 |
| F-412 | St. Louis County | *Chrysops* | *Elaeophora schneideri* | ES-1 | KT885226 |
| F-414 | St. Louis County | *Chrysops* | *Elaeophora schneideri* | ES-1 | KT885226 |
| F-415 | St. Louis County | *Chrysops* | *Elaeophora schneideri* | ES-1 | KT885226 |
| F-416 | St. Louis County | *Chrysops* | *Elaeophora schneideri* | ES-1 | KT885226 |
| F-411 | St. Louis County | *Chrysops* | *Elaeophora schneideri* | ES-1 | KT885226 |
| F-407 | St. Louis County | *Chrysops* | *Elaeophora schneideri* | ES-1 | KT885226 |
| F-413 | St. Louis County | *Chrysops* | *Elaeophora schneideri* | **ES-2** | KT885227 |
| F-334 | Anoka County | *Chrysops* | *Elaeophora schneideri* | ES-1 | KT885226 |
| F-367 | Anoka County | *Chrysops* | *Elaeophora schneideri* | ES-1 | KT885226 |
| F-368 | Anoka County | *Chrysops* | *Elaeophora schneideri* | ES-1 | KT885226 |
| F-369 | Anoka County | *Chrysops* | *Elaeophora schneideri* | **ND** | **ND** |
| F-371 | Anoka County | *Chrysops* | *Elaeophora schneideri* | ES-1 | KT885226 |
| F-372 | Anoka County | *Hybomitra* | *Elaeophora schneideri* | ES-1 | KT885226 |
| F-380 | Anoka County | *Hybomitra* | *Elaeophora schneideri* | ES-1 | KT885226 |
| F-382 | Anoka County | *Hybomitra* | *Elaeophora schneideri* | ES-1 | KT885226 |
| F-386 | Anoka County | *Hybomitra* | *Elaeophora schneideri* | ES-1 | KT885226 |
| F-383 | Anoka County | *Hybomitra* | *Elaeophora schneideri* | ES-1 | KT885226 |
| F-419 | Lake County | *Chrysops* | *Elaeophora schneideri* | ES-1 | KT885226 |
| F-422 | Lake County | *Chrysops* | *Elaeophora schneideri* | ES-1 | KT885226 |
| F-416 | Lake County | *Chrysops* | *Elaeophora schneideri* | ES-1 | KT885226 |
| F-417 | Lake County | *Chrysops* | *Elaeophora schneideri* | ES-1 | KT885226 |
| F-420 | Lake County | *Chrysops* | *Elaeophora schneideri* | ES-1 | KT885226 |
| F-421 | Lake County | *Chrysops* | *Elaeophora schneideri* | ES-1 | KT885226 |
| F-411 | Lake County | *Chrysops* | *Elaeophora schneideri* | ES-1 | KT885226 |
| F-556 | Grand Portage | *Chrysops* | Unknown Filarid spp. | NA | KT907501 |
| F-571 | Grand Portage | *Chrysops* | Unknown Filarid spp. | NA | KT907502 |
| F-555 | Grand Portage | *Chrysops* | Unknown Filarid spp. | NA | KT907503 |
